# Nasal Reconstruction After Basal Cell Excision

**Published:** 2013-01-21

**Authors:** Brenon L Abernathie, Mark Granick

**Affiliations:** Division of Plastic Surgery, University of Medicine and Dentistry of New Jersey, Newark

**Figure:**
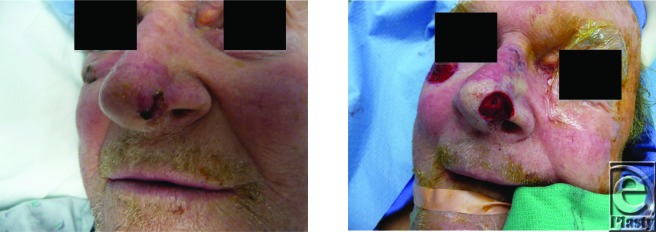


## DESCRIPTION

A 68-year-old man presents with growing lesion involving the nasal tip and ala.

## QUESTIONS

1. **What is the differential diagnosis for malignant lesions on the skin of the nose?**
2. **What are the principles of nasal reconstruction?**
3. **What reconstructive options are used to repair nasal defects?**

## DISCUSSION

The differential diagnosis for malignant lesions of the nose includes basal cell carcinoma, squamous cell carcinoma, and melanoma. Excessive sun exposure, increased age, and low Fitzpatrick skin types are shared risk factors for these malignancies. While atypical presentations may exist, these lesions often have distinct differences in appearance. Basal cells are classically described as having a pearly, rolled border with telangiectasias and possible central depression or pit. Squamous cell carcinomas often form a central area of ulceration or rough, crust-like appearance. Asymmetrical lesions with irregular borders and variegated colors are typical of melanoma. Other malignant lesions may occur on the nose, but these 3 are the most common.

The nose occupies a prominent place in the center of the face, making it a structure of obvious aesthetic significance, and its anatomy can be viewed as consisting of 9 subunits: dorsum, tip, columella, paired sidewalls, paired ala, and paired soft triangles. As a general rule of nasal reconstruction, if a defect encompasses more than 50% of a subunit, then the entire subunit should be reconstructed. It is often preferable to place incisions at the borders of subunits for better scar concealment and replace tissue with similar color, thickness, and texture.

Reconstructive options range from primary closure and skin grafting to local, axial (regional), or even composite (free) flap reconstruction. The method chosen is dependent on the defect's size, location, and structural involvement (ie, skin, cartilage, bone, mucosa). In the case described earlier, after negative margins were achieved on frozen section following excision of this patient's basal cell carcinoma, a dorsonasal flap was created. This is a skin transposition flap utilizing the dorsal nasal skin, which provides a good color and texture match for nasal reconstruction. Dorsonasal flaps may be useful in closing defects of less than 2 cm in diameter at the distal end of the nose, optimally with 0.5 cm of retained alar rim. After incising the flap, the entire nasal surface should be undermined at the level of the periosteum or perichondrium to aid in preservation of its blood supply from the angular artery. One disadvantage to this flap is the potential for cephalic displacement of the tip and/or ala should excess tension be required to adequately close the defect.

## Figures and Tables

**Figure:**
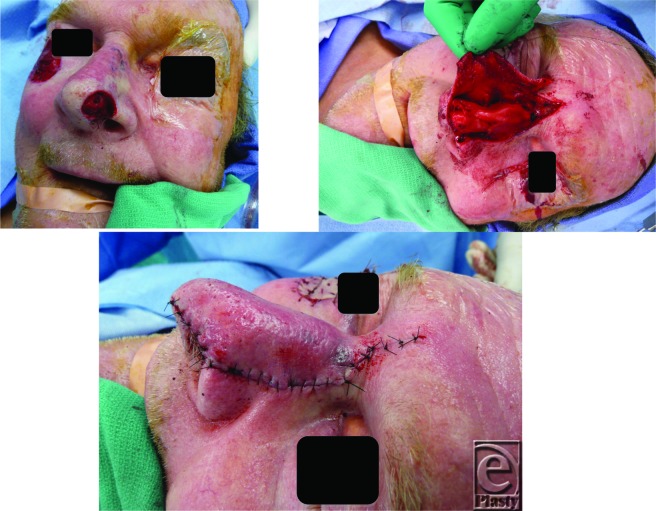
a. Defect following frozen section confirmation of margins. b. Dorsonasal flap raised. c. Flap inset.
